# 4-But­oxy-3-(2,4-dichloro­phen­yl)-1-oxaspiro­[4.5]dec-3-en-2-one

**DOI:** 10.1107/S1600536809000579

**Published:** 2009-01-14

**Authors:** Liang-zhong Xu, Shan-qi Sun, Wei Guo, Qun-qun Su

**Affiliations:** a College of Chemistry and Molecular Engineering, Qingdao University of Science and Technology, Qingdao 266042, People’s Republic of China

## Abstract

In the title compound, C_19_H_22_Cl_2_O_3_, the cyclo­hexane ring adopts a chair conformation. The furan ring plane forms dihedral angles of 81.88 (2) and 50.19 (3)°, respectively, with the benzene ring and the plane formed by the butyl C atoms. The crystal structure is stabilized by weak inter­molecular C—H⋯O hydrogen bonds.

## Related literature

For the biological activity of related compounds, see: Thomas *et al.* (2003 ▶). For synthetic information, see: Raeppel *et al.* (1998 ▶); Sarcevic *et al.* (1973 ▶).
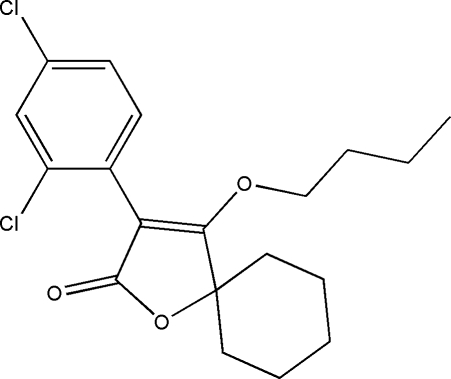


## Experimental

### 

#### Crystal data

C_19_H_22_Cl_2_O_3_
*M* *_r_* = 369.27
Orthorhombic, 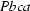

*a* = 15.177 (3) Å
*b* = 13.735 (3) Å
*c* = 17.497 (4) Å
*V* = 3647.4 (13) Å^3^
*Z* = 8
Mo *K*α radiation
μ = 0.37 mm^−1^
*T* = 113 (2) K
0.18 × 0.14 × 0.10 mm

#### Data collection

Rigaku Saturn diffractometer
Absorption correction: multi-scan (*CrystalClear*; Rigaku, 2005 ▶) *T*_min_ = 0.936, *T*_max_ = 0.964
23387 measured reflections
3217 independent reflections
2536 reflections with *I* > 2σ(*I*)
*R*_int_ = 0.090

#### Refinement

*R*[*F* ^2^ > 2σ(*F* ^2^)] = 0.045
*wR*(*F* ^2^) = 0.131
*S* = 1.08
3217 reflections
218 parameters
H-atom parameters constrained
Δρ_max_ = 0.60 e Å^−3^
Δρ_min_ = −0.35 e Å^−3^

### 

Data collection: *CrystalClear* (Rigaku, 2005 ▶); cell refinement: *CrystalClear*; data reduction: *CrystalClear*; program(s) used to solve structure: *SHELXTL* (Sheldrick, 2008 ▶); program(s) used to refine structure: *SHELXTL*; molecular graphics: *SHELXTL*; software used to prepare material for publication: *SHELXTL*.

## Supplementary Material

Crystal structure: contains datablocks I, global. DOI: 10.1107/S1600536809000579/lh2747sup1.cif

Structure factors: contains datablocks I. DOI: 10.1107/S1600536809000579/lh2747Isup2.hkl

Additional supplementary materials:  crystallographic information; 3D view; checkCIF report

## Figures and Tables

**Table 1:** Hydrogen-bond geometry (Å, °)

*D*—H⋯*A*	*D*—H	H⋯*A*	*D*⋯*A*	*D*—H⋯*A*
C2—H2*B*⋯O2^i^	0.97	2.54	3.316 (3)	137
C18—H18⋯O1^ii^	0.93	2.49	3.370 (3)	159

Symmetry codes: (i) 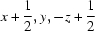; (ii) 
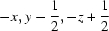
.

**Fig. 1.**
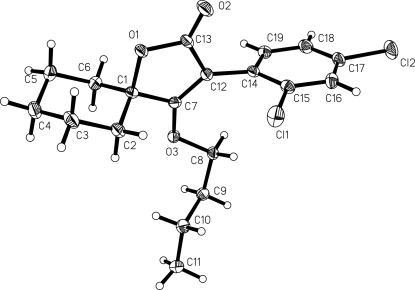
The molecular structure of (I), with displacement ellipsoids drawn at the 40% probability level.

## supplementary crystallographic information

### Comment

The title compound (I) was prepared as part of a project in search for new compounds with biological activity (Thomas *et al.*, 2003). We report here the crystal structure of (I).

In (I) (Fig. 1), all bond lengths and angles are normal and in a good agreement with those reported previously (Thomas *et al.*, 2003). The cyclohexane ring (C1—C6) adopts a chair conformation. The furan ring (O1/C1/C7/C12/C13) plane forms dihedral angles of 81.88 (2)° and 50.19 (3)° with the benzene ring (C14—C19) and the butyl group plane (C8—C11) respectively. In addition to van der Waals forces, the structure is stabilized by weak  C—H···O hydrogen bonds.

### Experimental

3-(2,4-Dichlorophenyl)-2,4-dioxo-1-oxaspiro[4.5]decane 3.13 g (10.0 mmol), was suspended in a solution of sodium carbonate 0.54 g (5.1 mmol) in 20 ml of water in a flask equipped with stirrer, water separator and reflux condenser. Toluene (40 ml) was added after 0.5 h, the mixture was heated to dehydration to distill the toluene solvent. Then 1-bromobutane 1.51 g (11.0 mmol) and *N*,*N*-dimethylformamide(DMF) solvent (20 ml) were added while maintaining the temperature at 373K for 4 h. Upon cooling at room temperature water (20 ml) was added. The mixture was extracted with CH_2_Cl_2_ (15 ml) and the organic layer was washed with water and dried over sodium sulfate. The excess CH_2_Cl_2_ was removed on a water vacuum pump to obtain the oily product which was crystallized from methanol to afford the title compound 2.95 g (80% yield) (Raeppel *et al.*, 1998; Sarcevic *et al.*, 1973). Single crystals suitable for X-ray diffraction were obtained by recrystallization of the title compound from a mixture of acetone and methanol at room temperature.

### Refinement

All C-bound H atoms were placed in calculated positions, with C—H = 0.93–0.97 Å, and included in the final cycles of refinement using a riding model, with *U*_iso_(H) = 1.2*U*_eq_(C) for the aryl and methylene H atoms and 1.5*U*_eq_(C) for the methyl H atoms.

### Crystal data

**Table:**

C_19_H_22_Cl_2_O_3_	*F*(000) = 1552
*M**_r_* = 369.27	*D*_x_ = 1.345 Mg m^−^^3^
Orthorhombic, *P**b**c**a*	Mo *K*α radiation, λ = 0.71073 Å
Hall symbol: -P 2ac 2ab	Cell parameters from 7182 reflections
*a* = 15.177 (3) Å	θ = 2.3–27.5°
*b* = 13.735 (3) Å	µ = 0.37 mm^−^^1^
*c* = 17.497 (4) Å	*T* = 113 K
*V* = 3647.4 (13) Å^3^	Platelet, colorless
*Z* = 8	0.18 × 0.14 × 0.10 mm

### Data collection

**Table:**

Rigaku Saturn diffractometer	3217 independent reflections
Radiation source: rotating anode	2536 reflections with *I* > 2σ(*I*)
confocal	*R*_int_ = 0.090
ω scans	θ_max_ = 25.0°, θ_min_ = 2.3°
Absorption correction: multi-scan (*CrystalClear*; Rigaku, 2005)	*h* = −17→18
*T*_min_ = 0.936, *T*_max_ = 0.964	*k* = −16→13
23387 measured reflections	*l* = −20→19

### Refinement

**Table:**

Refinement on *F*^2^	Primary atom site location: structure-invariant direct methods
Least-squares matrix: full	Secondary atom site location: difference Fourier map
*R*[*F*^2^ > 2σ(*F*^2^)] = 0.045	Hydrogen site location: inferred from neighbouring sites
*wR*(*F*^2^) = 0.131	H-atom parameters constrained
*S* = 1.08	*w* = 1/[σ^2^(*F*_o_^2^) + (0.0705*P*)^2^ + 0.1293*P*] where *P* = (*F*_o_^2^ + 2*F*_c_^2^)/3
3217 reflections	(Δ/σ)_max_ = 0.001
218 parameters	Δρ_max_ = 0.60 e Å^−^^3^
0 restraints	Δρ_min_ = −0.35 e Å^−^^3^

### Special details

**Table:**

Geometry. All e.s.d.'s (except the e.s.d. in the dihedral angle between two l.s. planes) are estimated using the full covariance matrix. The cell e.s.d.'s are taken into account individually in the estimation of e.s.d.'s in distances, angles and torsion angles; correlations between e.s.d.'s in cell parameters are only used when they are defined by crystal symmetry. An approximate (isotropic) treatment of cell e.s.d.'s is used for estimating e.s.d.'s involving l.s. planes.
Refinement. Refinement of *F*^2^ against ALL reflections. The weighted *R*-factor *wR* and goodness of fit *S* are based on *F*^2^, conventional *R*-factors *R* are based on *F*, with *F* set to zero for negative *F*^2^. The threshold expression of *F*^2^ > σ(*F*^2^) is used only for calculating *R*-factors(gt) *etc*. and is not relevant to the choice of reflections for refinement. *R*-factors based on *F*^2^ are statistically about twice as large as those based on *F*, and *R*- factors based on ALL data will be even larger.

### Fractional atomic coordinates and isotropic or equivalent isotropic displacement parameters (Å^2^)

**Table:**

	*x*	*y*	*z*	*U*_iso_*/*U*_eq_	
Cl1	0.10283 (4)	0.81909 (5)	0.06309 (3)	0.0362 (2)	
Cl2	−0.13817 (4)	0.55405 (6)	−0.02124 (4)	0.0402 (2)	
O1	0.12744 (9)	0.83740 (13)	0.31227 (8)	0.0267 (4)	
O2	−0.00361 (10)	0.84602 (15)	0.25290 (10)	0.0382 (5)	
O3	0.24825 (9)	0.64650 (13)	0.22585 (8)	0.0234 (4)	
C1	0.20839 (12)	0.78176 (19)	0.30321 (12)	0.0211 (5)	
C2	0.28018 (14)	0.84967 (19)	0.27409 (13)	0.0244 (5)	
H2A	0.2608	0.8795	0.2267	0.029*	
H2B	0.3328	0.8120	0.2632	0.029*	
C3	0.30279 (14)	0.9295 (2)	0.33171 (13)	0.0299 (6)	
H3A	0.3507	0.9690	0.3121	0.036*	
H3B	0.2521	0.9714	0.3391	0.036*	
C4	0.32958 (15)	0.8847 (2)	0.40787 (13)	0.0323 (6)	
H4A	0.3401	0.9362	0.4447	0.039*	
H4B	0.3841	0.8489	0.4014	0.039*	
C5	0.25880 (15)	0.8165 (2)	0.43855 (13)	0.0307 (6)	
H5A	0.2067	0.8540	0.4513	0.037*	
H5B	0.2799	0.7859	0.4850	0.037*	
C6	0.23407 (14)	0.7375 (2)	0.38050 (12)	0.0259 (6)	
H6A	0.1851	0.6997	0.4002	0.031*	
H6B	0.2837	0.6938	0.3735	0.031*	
C7	0.18423 (13)	0.70785 (18)	0.24397 (12)	0.0195 (5)	
C8	0.23904 (13)	0.58949 (18)	0.15675 (12)	0.0225 (5)	
H8A	0.2200	0.6304	0.1147	0.027*	
H8B	0.1959	0.5382	0.1640	0.027*	
C9	0.32863 (14)	0.54638 (17)	0.13996 (13)	0.0241 (5)	
H9A	0.3247	0.5073	0.0939	0.029*	
H9B	0.3450	0.5035	0.1816	0.029*	
C10	0.40051 (13)	0.6221 (2)	0.12948 (13)	0.0275 (6)	
H10A	0.4078	0.6583	0.1767	0.033*	
H10B	0.3829	0.6677	0.0899	0.033*	
C11	0.48797 (15)	0.5757 (2)	0.10750 (14)	0.0380 (7)	
H11A	0.5013	0.5239	0.1425	0.057*	
H11B	0.5338	0.6238	0.1096	0.057*	
H11C	0.4840	0.5501	0.0566	0.057*	
C12	0.10200 (13)	0.72226 (19)	0.21741 (12)	0.0204 (5)	
C13	0.06647 (13)	0.80578 (19)	0.25970 (12)	0.0255 (6)	
C14	0.04851 (13)	0.67432 (18)	0.15677 (12)	0.0207 (5)	
C15	0.04096 (13)	0.71635 (18)	0.08456 (12)	0.0229 (5)	
C16	−0.01452 (14)	0.67937 (18)	0.02864 (12)	0.0257 (5)	
H16	−0.0185	0.7085	−0.0192	0.031*	
C17	−0.06371 (14)	0.59783 (19)	0.04641 (13)	0.0267 (6)	
C18	−0.05625 (14)	0.5511 (2)	0.11608 (13)	0.0285 (6)	
H18	−0.0881	0.4947	0.1262	0.034*	
C19	−0.00021 (13)	0.59027 (18)	0.17061 (12)	0.0240 (5)	
H19	0.0050	0.5595	0.2178	0.029*	

### Atomic displacement parameters (Å^2^)

**Table:**

	*U*^11^	*U*^22^	*U*^33^	*U*^12^	*U*^13^	*U*^23^
Cl1	0.0475 (4)	0.0282 (4)	0.0329 (4)	−0.0146 (3)	−0.0045 (3)	0.0049 (3)
Cl2	0.0339 (3)	0.0451 (5)	0.0417 (4)	−0.0047 (3)	−0.0147 (3)	−0.0141 (3)
O1	0.0154 (7)	0.0332 (11)	0.0316 (9)	0.0046 (7)	−0.0038 (6)	−0.0143 (8)
O2	0.0195 (8)	0.0453 (13)	0.0499 (11)	0.0089 (8)	−0.0087 (7)	−0.0205 (10)
O3	0.0208 (7)	0.0267 (10)	0.0229 (8)	0.0088 (7)	−0.0027 (6)	−0.0074 (7)
C1	0.0151 (9)	0.0250 (13)	0.0231 (11)	0.0041 (11)	−0.0005 (8)	−0.0033 (10)
C2	0.0188 (10)	0.0277 (14)	0.0269 (12)	−0.0003 (11)	−0.0027 (8)	0.0018 (11)
C3	0.0214 (11)	0.0281 (14)	0.0402 (14)	−0.0038 (11)	−0.0049 (9)	−0.0051 (12)
C4	0.0254 (11)	0.0382 (16)	0.0334 (12)	−0.0002 (12)	−0.0081 (10)	−0.0073 (12)
C5	0.0309 (12)	0.0395 (16)	0.0215 (12)	0.0013 (12)	−0.0045 (9)	−0.0062 (11)
C6	0.0252 (11)	0.0301 (15)	0.0224 (11)	−0.0021 (11)	0.0006 (8)	−0.0013 (11)
C7	0.0180 (10)	0.0211 (12)	0.0195 (10)	0.0015 (11)	0.0022 (8)	0.0001 (9)
C8	0.0202 (10)	0.0250 (14)	0.0224 (11)	0.0031 (10)	−0.0031 (8)	−0.0061 (10)
C9	0.0232 (11)	0.0238 (13)	0.0254 (11)	0.0027 (11)	0.0010 (8)	−0.0069 (10)
C10	0.0255 (11)	0.0309 (15)	0.0259 (11)	0.0015 (12)	−0.0007 (9)	0.0018 (11)
C11	0.0247 (11)	0.062 (2)	0.0269 (12)	−0.0027 (14)	0.0036 (9)	−0.0034 (13)
C12	0.0186 (10)	0.0220 (13)	0.0206 (11)	0.0014 (11)	−0.0015 (8)	−0.0020 (10)
C13	0.0142 (10)	0.0326 (15)	0.0298 (12)	−0.0007 (11)	−0.0032 (8)	−0.0091 (11)
C14	0.0149 (9)	0.0240 (12)	0.0231 (11)	0.0029 (10)	−0.0006 (8)	−0.0048 (10)
C15	0.0198 (9)	0.0206 (12)	0.0284 (11)	−0.0028 (11)	−0.0005 (8)	−0.0025 (10)
C16	0.0289 (11)	0.0248 (14)	0.0233 (12)	0.0042 (11)	−0.0053 (9)	−0.0012 (10)
C17	0.0201 (10)	0.0293 (14)	0.0307 (12)	−0.0009 (11)	−0.0050 (9)	−0.0106 (11)
C18	0.0224 (11)	0.0292 (14)	0.0340 (13)	−0.0060 (11)	0.0004 (9)	−0.0034 (11)
C19	0.0214 (10)	0.0268 (14)	0.0239 (11)	−0.0031 (11)	0.0011 (8)	0.0013 (10)

### Geometric parameters (Å, °)

**Table:**

Cl1—C15	1.736 (2)	C7—C12	1.346 (3)
Cl2—C17	1.744 (2)	C8—C9	1.512 (3)
O1—C13	1.375 (3)	C8—H8A	0.9700
O1—C1	1.455 (2)	C8—H8B	0.9700
O2—C13	1.204 (3)	C9—C10	1.519 (3)
O3—C7	1.325 (3)	C9—H9A	0.9700
O3—C8	1.447 (3)	C9—H9B	0.9700
C1—C7	1.497 (3)	C10—C11	1.522 (3)
C1—C2	1.522 (3)	C10—H10A	0.9700
C1—C6	1.533 (3)	C10—H10B	0.9700
C2—C3	1.529 (3)	C11—H11A	0.9600
C2—H2A	0.9700	C11—H11B	0.9600
C2—H2B	0.9700	C11—H11C	0.9600
C3—C4	1.523 (3)	C12—C13	1.468 (3)
C3—H3A	0.9700	C12—C14	1.489 (3)
C3—H3B	0.9700	C14—C19	1.392 (3)
C4—C5	1.523 (4)	C14—C15	1.394 (3)
C4—H4A	0.9700	C15—C16	1.387 (3)
C4—H4B	0.9700	C16—C17	1.381 (3)
C5—C6	1.533 (3)	C16—H16	0.9300
C5—H5A	0.9700	C17—C18	1.382 (3)
C5—H5B	0.9700	C18—C19	1.387 (3)
C6—H6A	0.9700	C18—H18	0.9300
C6—H6B	0.9700	C19—H19	0.9300
			
C13—O1—C1	109.23 (17)	C9—C8—H8B	110.4
C7—O3—C8	118.25 (16)	H8A—C8—H8B	108.6
O1—C1—C7	103.00 (15)	C8—C9—C10	113.7 (2)
O1—C1—C2	108.6 (2)	C8—C9—H9A	108.8
C7—C1—C2	111.05 (17)	C10—C9—H9A	108.8
O1—C1—C6	109.07 (16)	C8—C9—H9B	108.8
C7—C1—C6	113.8 (2)	C10—C9—H9B	108.8
C2—C1—C6	110.88 (17)	H9A—C9—H9B	107.7
C1—C2—C3	112.31 (18)	C9—C10—C11	111.7 (2)
C1—C2—H2A	109.1	C9—C10—H10A	109.3
C3—C2—H2A	109.1	C11—C10—H10A	109.3
C1—C2—H2B	109.1	C9—C10—H10B	109.3
C3—C2—H2B	109.1	C11—C10—H10B	109.3
H2A—C2—H2B	107.9	H10A—C10—H10B	107.9
C4—C3—C2	110.3 (2)	C10—C11—H11A	109.5
C4—C3—H3A	109.6	C10—C11—H11B	109.5
C2—C3—H3A	109.6	H11A—C11—H11B	109.5
C4—C3—H3B	109.6	C10—C11—H11C	109.5
C2—C3—H3B	109.6	H11A—C11—H11C	109.5
H3A—C3—H3B	108.1	H11B—C11—H11C	109.5
C3—C4—C5	111.63 (18)	C7—C12—C13	106.35 (19)
C3—C4—H4A	109.3	C7—C12—C14	133.3 (2)
C5—C4—H4A	109.3	C13—C12—C14	120.29 (18)
C3—C4—H4B	109.3	O2—C13—O1	121.0 (2)
C5—C4—H4B	109.3	O2—C13—C12	129.3 (2)
H4A—C4—H4B	108.0	O1—C13—C12	109.68 (18)
C4—C5—C6	112.01 (19)	C19—C14—C15	117.2 (2)
C4—C5—H5A	109.2	C19—C14—C12	122.2 (2)
C6—C5—H5A	109.2	C15—C14—C12	120.5 (2)
C4—C5—H5B	109.2	C16—C15—C14	122.5 (2)
C6—C5—H5B	109.2	C16—C15—Cl1	118.24 (18)
H5A—C5—H5B	107.9	C14—C15—Cl1	119.23 (17)
C5—C6—C1	111.5 (2)	C17—C16—C15	117.8 (2)
C5—C6—H6A	109.3	C17—C16—H16	121.1
C1—C6—H6A	109.3	C15—C16—H16	121.1
C5—C6—H6B	109.3	C16—C17—C18	122.1 (2)
C1—C6—H6B	109.3	C16—C17—Cl2	118.49 (19)
H6A—C6—H6B	108.0	C18—C17—Cl2	119.44 (19)
O3—C7—C12	133.7 (2)	C17—C18—C19	118.5 (2)
O3—C7—C1	114.69 (17)	C17—C18—H18	120.8
C12—C7—C1	111.49 (19)	C19—C18—H18	120.8
O3—C8—C9	106.70 (16)	C18—C19—C14	121.9 (2)
O3—C8—H8A	110.4	C18—C19—H19	119.1
C9—C8—H8A	110.4	C14—C19—H19	119.1
O3—C8—H8B	110.4		
			
C13—O1—C1—C7	−5.1 (2)	O3—C7—C12—C14	0.6 (5)
C13—O1—C1—C2	112.8 (2)	C1—C7—C12—C14	176.3 (2)
C13—O1—C1—C6	−126.3 (2)	C1—O1—C13—O2	−174.7 (2)
O1—C1—C2—C3	64.3 (2)	C1—O1—C13—C12	4.5 (3)
C7—C1—C2—C3	176.88 (19)	C7—C12—C13—O2	177.3 (3)
C6—C1—C2—C3	−55.5 (3)	C14—C12—C13—O2	−0.8 (4)
C1—C2—C3—C4	56.3 (2)	C7—C12—C13—O1	−1.9 (3)
C2—C3—C4—C5	−55.4 (3)	C14—C12—C13—O1	−179.97 (19)
C3—C4—C5—C6	54.8 (3)	C7—C12—C14—C19	84.9 (3)
C4—C5—C6—C1	−53.5 (3)	C13—C12—C14—C19	−97.6 (3)
O1—C1—C6—C5	−66.1 (2)	C7—C12—C14—C15	−99.4 (3)
C7—C1—C6—C5	179.54 (18)	C13—C12—C14—C15	78.1 (3)
C2—C1—C6—C5	53.5 (2)	C19—C14—C15—C16	2.1 (3)
C8—O3—C7—C12	10.9 (4)	C12—C14—C15—C16	−173.8 (2)
C8—O3—C7—C1	−164.6 (2)	C19—C14—C15—Cl1	−178.01 (16)
O1—C1—C7—O3	−179.45 (18)	C12—C14—C15—Cl1	6.0 (3)
C2—C1—C7—O3	64.5 (3)	C14—C15—C16—C17	0.1 (3)
C6—C1—C7—O3	−61.5 (2)	Cl1—C15—C16—C17	−179.75 (17)
O1—C1—C7—C12	4.0 (2)	C15—C16—C17—C18	−2.6 (4)
C2—C1—C7—C12	−112.1 (2)	C15—C16—C17—Cl2	176.63 (17)
C6—C1—C7—C12	122.0 (2)	C16—C17—C18—C19	2.8 (4)
C7—O3—C8—C9	166.18 (19)	Cl2—C17—C18—C19	−176.49 (18)
O3—C8—C9—C10	−58.8 (2)	C17—C18—C19—C14	−0.4 (4)
C8—C9—C10—C11	−176.20 (18)	C15—C14—C19—C18	−2.0 (3)
O3—C7—C12—C13	−177.1 (2)	C12—C14—C19—C18	173.9 (2)
C1—C7—C12—C13	−1.5 (3)		

### Hydrogen-bond geometry (Å, °)

**Table:**

*D*—H···*A*	*D*—H	H···*A*	*D*···*A*	*D*—H···*A*
C2—H2B···O2^i^	0.97	2.54	3.316 (3)	137
C18—H18···O1^ii^	0.93	2.49	3.370 (3)	159

Symmetry codes: (i) *x*+1/2, *y*, −*z*+1/2; (ii) −*x*, *y*−1/2, −*z*+1/2.
